# Weight and metabolic changes in patients with schizophrenia treated with paliperidone palmitate 6-month formulation versus paliperidone palmitate 3-month formulation: a post-hoc analysis

**DOI:** 10.1093/ijnp/pyag042

**Published:** 2026-08-20

**Authors:** Mark Guirguis, Robert Karl Knight, Panna Sanga, John Han, Silvana Galderisi

**Affiliations:** Johnson & Johnson Innovative Medicine, LLC, Titusville, NJ, United States; Johnson & Johnson Innovative Medicine, LLC, Titusville, NJ, United States; Johnson & Johnson Innovative Medicine, LLC, Titusville, NJ, United States; Johnson & Johnson Innovative Medicine, LLC, Titusville, NJ, United States; University of Campania Luigi Vanvitelli, Naples, Italy

**Keywords:** schizophrenia, paliperidone palmitate, weight gain, body mass index, long-acting injectable

## Abstract

**Objective:**

To determine the effect that paliperidone palmitate 6-month long-acting injectable formulation (PP6M) had on metabolic parameters including body weight (BW), and blood lipid profiles, a post-hoc analysis was conducted to assess changes in BW from baseline to the end of study based on age, body mass index (BMI), and changes in blood lipid profiles during a 12-month, phase 3, double-blind (DB) clinical study. Long term effects of PP6M on BW and BMI were further explored during a 24-month extension study in which participants were treated exclusively with PP6M.

**Method:**

In the 12-month DB phase, participants were randomized to receive PP6M or paliperidone palmitate 3-month long-acting injectable formulation (PP3M). The mean change in BW and abnormal weight percent change from baseline were calculated at endpoint by age, gender, and BMI. Additionally, treatment-emergent shifts from baseline for the four key lipid parameters (fasting low density lipoprotein [LDL], fasting triglycerides [TG], fasting total cholesterol [TC], and fasting high density lipoprotein [HDL]) during DB were assessed. Following this study, participants were given the opportunity to transition to a 24-month extension study and be treated with PP6M. The mean change and percent change in BW, and the mean change in BMI from DB baseline to the end of the extension study (36 months total) were calculated.

**Results:**

Participants who were treated with PP6M showed numerically less weight gain, BMI, waist circumference, and more weight decrease compared to PP3M group during 12-month DB phase, though the proportion of participants reporting an abnormal change (≥7% change) in BW did not significantly differ between PP3M and PP6M. The weight differences were more pronounced in the younger age group (18-25 years) and those who were overweight (BMI: 25 to <30 kg/m^2^. Numerical differences in favor of PP6M were found in fasting blood lipids (HDL, LDL, TG, and TC). The changes in BW and BMI over time remained consistent throughout the 24-month extension, favoring PP6M in each instance.

**Conclusions:**

This post-hoc analysis demonstrated that PP6M was comparable to PP3M in terms of metabolic parameters; however, it may have a beneficial effect on weight gain, especially in young patients.

**Trial registration:**

Post-Hoc Analysis of Studies NCT03345342 and NCT04072575 (ClinicalTrials.gov).

**Significant outcomes**

- The findings from this study have highlighted that participants who were treated with the 6-month long-acting injectable formulation of paliperidone exhibited less weight gain during treatment overall, and significantly less weight gain in adolescents and young adults. Importantly, this trend continued over the course of long-term treatment, regardless of age. Participants treated with the 6-month formulation also had fewer shifts in blood lipids outside of the normal range and had more favorable changes in body mass index and waist circumference. These results suggest that when considering metabolic dysregulation as a factor in choosing a long-acting injectable antipsychotic, the 6-month formulation is a viable alternative to the 3-month formulation, particularly in younger patients with schizophrenia.

**Limitations**

- Because this is a post hoc analysis and the study was not powered to test weight and metabolic changes, most endpoints were summarized descriptively and the statistical test was limited to the main endpoint (abnormal percent weight gain and loss).

## Introduction

Weight gain and metabolic dysregulation are prevalent in patients with schizophrenia and are well-documented adverse events (AEs) associated with antipsychotic use. Patients with schizophrenia have double the all-cause mortality rate compared to the general population, due to a multitude of factors that include increased risks of cardiovascular disease, respiratory disease (due primarily to high rates of smoking), some cancers, suicide, accidents, and homicide. The increased rate of obesity and elevated lipid levels, that are commonly associated with antipsychotic use^1^^,^^2^ add to the overall cardiovascular risk in these patients, and the management of this metabolic dysregulation adds to the overall disease burden associated with schizophrenia.^3^ Given the significant disease burden, medications that contribute to weight gain further exacerbate this already substantial challenge. It is well documented that long-acting injectable (LAI) antipsychotics increase drug adherence resulting in an ultimately better outcome for patients with schizophrenia.^4^ However, as with oral antipsychotics, LAIs have been associated with high rates of metabolic syndrome^5–8^ and substantial weight increases over time.^5^^,^^9–14^ Importantly, a significant dose–response association for weight and metabolic variables in patients treated with oral or LAI antipsychotics has been reported, with each antipsychotic having a dose–response profile.^15^^,^^16^

Several studies have shown that metabolic syndrome (including weight gain) has been observed with both first-generation and second-generation LAI antipsychotics.^6^^,^^7^^,^^17^^,^^18^ One study showed that LAI formulations of second-generation antipsychotics (LAI SGAs) were associated with more positive results in terms of metabolic syndrome criteria than oral SGA.^19^ A United States Food and Drug Administration AE Reporting System (FAERS) and Japanese Adverse Drug Event Report (JADER) data mining study^20^ identified weight gain as a persistent adverse reaction associated with paliperidone palmitate, with women more affected than men; the median onset time for increased weight was 121 days. One study showed that monotherapy with paliperidone palmitate was associated with a reduced risk of metabolic syndrome over time.^21^

Consistent with reports from adults, metabolic dysregulation in the form of weight gain was consistently reported during paliperidone palmitate use in the adolescent population.^22^^,^^23^ A retrospective analysis of efficacy and tolerability of paliperidone palmitate in treatment-naïve adolescents with first-episode schizophrenia reported that 1 out of 18 patients taking paliperidone palmitate experienced a metabolic treatment-emergent AE (TEAE) of weight gain during the 12-month study period.^22^ Importantly, a longitudinal study that monitored metabolic parameters in patients taking risperidone or paliperidone (oral or depot) for up to 1 year reported that the daily dosing interval had an increasing impact on weight change over 6 and 12 months in adolescents. Specifically, for risperidone, a dose effect on weight gain for daily dosing interval > 3 mg/d was detected, meaning that for each mg increase (eg, 3-4 mg/d), weight increased by 0.16%. On the other hand, for daily dosing intervals ≤3 mg/d, a dose effect was observed only at 3, 6, and 12. Thus, at 3, 6, and 12 months, higher weight gain estimates were reported for daily dosing intervals ≤3 mg/d as compared with higher doses, meaning that increasing daily dosing intervals from 1 mg to 2 mg/d led to a greater difference in weight gain than increasing from 5 to 6 mg/d.^23^

Two studies evaluated the safety and tolerability of paliperidone extended-release (ER) in children and adolescents. Savitz^24^ conducted a 2-year, open-label (OL), multicenter study in adolescents (12-17 years of age) with schizophrenia who were flexibly dosed with paliperidone ER (range: 1.5-12 mg/day). Weight increase was reported as a TEAE in 73 (18.3%) participants, 4 of which were considered severe, and 17.2% and 17.5% had clinically meaningful increases in body weight (BW) and body mass index (BMI), respectively. Joshi^25^ conducted an 8-week prospective, OL study to evaluate safety and efficacy of paliperidone monotherapy in 15 children and adolescents with bipolar spectrum disorders. In this population, 6 participants developed clinically significant weight gain (≥7%) following paliperidone therapy, and across all participants, weight gain at endpoint ranged from 0 to 12.5 lbs.

Given the clinical significance of metabolic dysregulation, understanding the metabolic effects of LAI antipsychotics with different dosing intervals (ie, paliperidone palmitate 1-month injectable [PP1M] versus paliperidone palmitate 3-month injectable [PP3M] versus paliperidone palmitate 6-month injectable [PP6M]), as well as broader trends in antipsychotic-induced weight gain and metabolic outcomes in adult and adolescent patients with schizophrenia is important. To determine the effect that PP6M had on metabolic parameters including BW, and blood lipid profiles, a post-hoc analysis was conducted to assess changes in BW from baseline to the end of study based on age, BMI, and changes in blood lipid profiles during a 12-month, phase 3, double-blind (DB) clinical study. The phase 3 DB study (NCT03345342) evaluated the efficacy and safety of PP6M LAI versus PP3M LAI in patients with schizophrenia. The study demonstrated that PP6M was non-inferior to PP3M in preventing relapses, with no new safety concerns.^26^ Long-term effects of PP6M on BW and BMI were further explored during a 24-month extension study in which participants were treated exclusively with PP6M. The results of this post-hoc analysis and its implications on the prescribed use of 3- and 6-month LAIs are also discussed. Full results have been published previously.^27^

## Data and methods

The study protocol, and amendments were approved by an independent ethics committee or institutional review board. The study was conducted in compliance with the ethical principles of the Declaration of Helsinki, Good Clinical Practices, and applicable regulatory requirements. All participants provided written informed consent before study participation. Participants enrolled in this study were required to meet the key entry criteria.

### Participants

The target population consisted of adult men and women aged 18-70 years (inclusive) who had a Diagnostic and Statistical Manual for Mental Disorders - 5th edition (DSM-5) diagnosis of schizophrenia for at least 6 months before screening and had a full Positive and Negative Symptom Score (PANSS) score of <70 points at screening. Participants were required to have received treatment with paliperidone palmitate (either the PP1M or PP3M formulation), injectable risperidone, or any oral antipsychotic. If the treatment was paliperidone palmitate, then the dose strength was required to be PP1M as 100 or 150 mg eq. or PP3M as 350 or 525 mg eq. and the dose timing had to fit the study schedule. If the treatment was injectable risperidone, then the dose strength had to be 50 mg, the dosing cycle had to be every 2 weeks, the efficacy and tolerability must have been established as adequate with the same strength and frequency for at least 3 injection cycles before screening, and the participant preferred a longer-acting injectable medication. If the treatment was an oral antipsychotic, then the participant must have had a valid reason to discontinue it, such as problems with efficacy, safety, or tolerability, or preferred a LAI medication. Eligible participants were required to be able, in the opinion of the investigator, to discontinue any antipsychotic medication other than PP1M or PP3M during the screening phase.

Exclusion criteria included receipt of any form of involuntary treatment, such as involuntary psychiatric hospitalization, parole-mandated treatment, or court-mandated treatment; suicide attempt within 12 months of screening and at imminent risk of suicide or violent behavior (as assessed by the investigator at screening); a DSM-5 diagnosis of moderate or severe substance use disorder (except for nicotine and caffeine) within 6 months of screening; history of neuroleptic malignant syndrome or tardive dyskinesia; treatment with LAI formulations of neuroleptic drugs other than risperidone or paliperidone (eg, haloperidol decanoate, fluphenazine decanoate) within 6 months of screening; primary, active DSM-5 psychiatric diagnosis other than schizophrenia; clinically significant and unstable medical illness in history or at screening that could interfere with the interpretation of the efficacy or safety measurements; any clinically relevant abnormality in the physical examination, vital signs, or 12-lead electrocardiogram (ECG) at screening; any clinically significant abnormal values for hematology, serum chemistry, or urinalysis at screening; and receipt of clozapine for treatment-resistant or treatment-refractory illness within 2 months of screening.

### Study design and objectives

**Figure 1:**
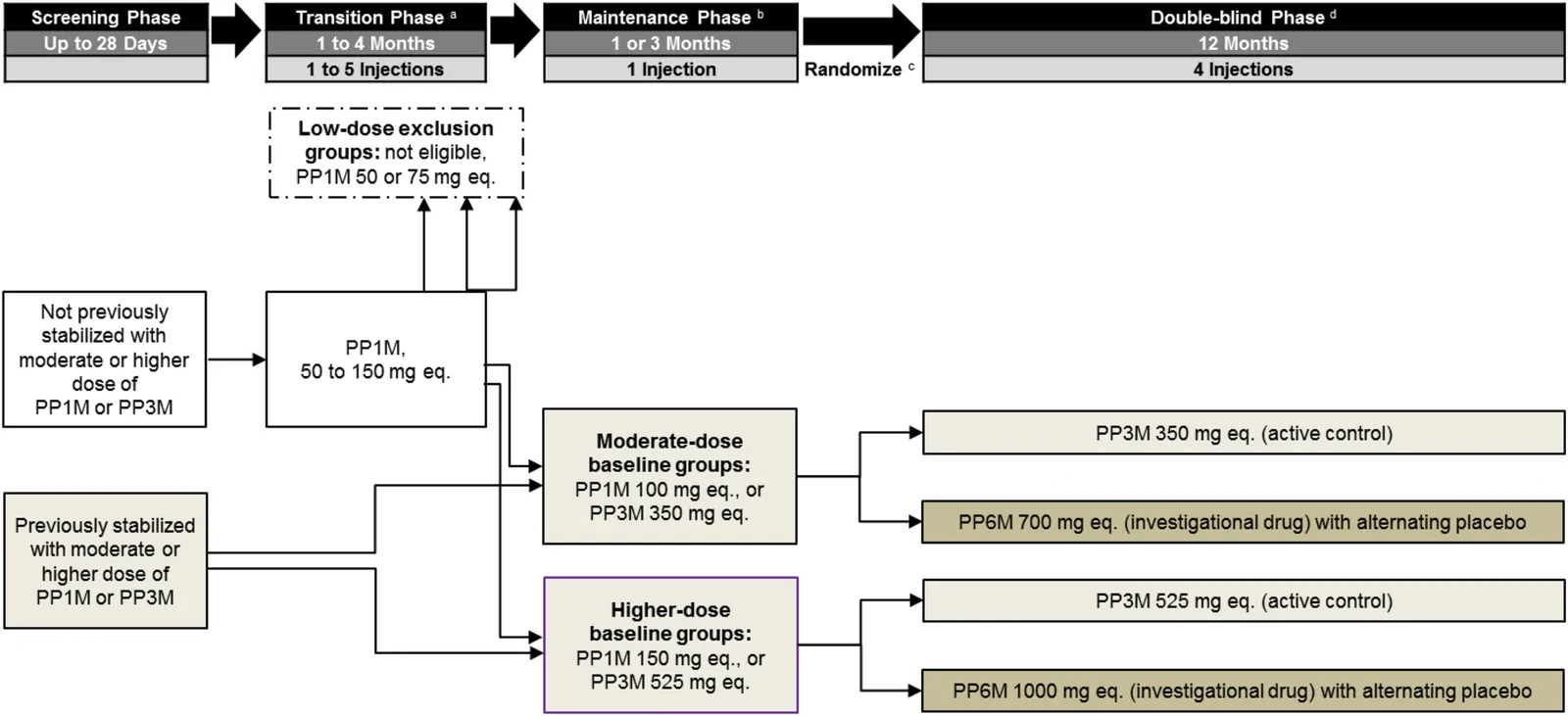
Study design.

This study (Protocol R092670PSY3015; NCT03345342) was a DB, randomized, active-controlled, parallel-group multicenter study conducted between November 2017 and May 2020 at 121 research sites in 20 countries. The study had 3 phases: (1) a screening phase (up to 28 days), (2) an OL maintenance phase (duration of 1 or 3 months depending on treatment received [1 injection cycle of PP1M/PP3M]), and (3) DB phase (12 months) (Figure 1). Participants who entered the study on an oral antipsychotic, injectable risperidone microspheres, or PP1M previously initiated but not stabilized at study entry were eligible to participate in an OL transition phase just prior to the OL maintenance phase to initiate and/or continue treatment with PP1M for up to 4 months. Participants on PP1M or PP3M OL maintenance treatment could be randomized to DB treatment with equivalent doses of PP6M or PP3M.

### Treatment

Study drugs were supplied in prefilled syringes and packaged in individual participant kits. Each kit consisted of a safety needle, instructions for use, and a blister-packed, prefilled syringe assembled with a plunger rod. All study agent labels contained information to meet the applicable regulatory requirements.

During the screening phase, enrolled participants had already received PP1M at 100 or 150 mg eq., PP3M at 350 or 525 mg eq., injectable risperidone at 50 mg, or an oral antipsychotic (at any dosage with a reason to change) as prior medications. The transition phase was applicable only to those participants who entered the screening phase without previous PP1M or PP3M stability. These participants may have been treated with oral antipsychotics, injectable risperidone, or a moderate or higher dose of PP1M with previous initiation but without previous stabilization (where stabilization was defined as at least 3 months of injections with the last 2 doses being the same strength). Participants who had been previously treated with oral antipsychotics received PP1M as 150 mg eq. on Day 1 and 100 mg eq. on Day 8. Thereafter, the PP1M doses on Days 36, 64, and 92 were flexible (as 50, 75, 100, or 150 mg eq.), based on clinical judgment and shared decision-making. Per the protocol, the 50 and 75 mg eq. doses were permitted at visits on Day 36 and Day 64, but use of these doses at these visits rendered the participants ineligible to proceed to the DB phase. Administration was in the deltoid muscle on Days 1 and 8 and was either in the deltoid or gluteal muscles on Days 36, 64, and 92. If a participant was previously treated with oral paliperidone ER tablets and had acceptable efficacy and tolerability, then the Day 36 dose of PP1M could be based on the conversion provided in protocol. If the previous dosage of oral paliperidone had insufficient efficacy, then the investigator could consider a higher dosage of PP1M. The investigator could consider a lower dose if the previous dosage of oral paliperidone caused problems with intolerability.

Participants who were previously stabilized on risperidone LAI were not required to attend the Day 1 visit. On the Day 8 visit, when the participant's next injectable risperidone dose was planned, PP1M 100 mg eq. was administered instead. Thereafter, the PP1M doses on Days 36, 64, and 92 were flexible (50, 75, 100, or 150 mg eq.), based on clinical judgment and shared decision-making. Administration was to be in the deltoid muscle on Day 8 and could be either in the deltoid or gluteal muscles on Days 36, 64, and 92.

For all participants, only 1 dose of PP1M as 100 or 150 mg eq. or PP3M as 350 or 525 mg eq was administered. Each participant’s maintenance phase dose was matched by straightforward progression (PP1M to PP1M, or PP3M to PP3M) or was matched by established conversion (PP1M to PP3M) to the same dose that they received during the screening phase or at the end of the transition phase. To ensure adequate numbers of participants treated with either PP1M or PP3M, some participants (after appropriate treatment with PP1M) were switched to PP3M during the maintenance phase as low enrollment of participants previously treated with PP3M was expected. This occurred, until the target (“PP3M pre-randomization target”) of approximately one-half of the total maintenance phase sample was treated with PP3M.

Throughout the study, participants who received PP3M as 350 or 525 mg eq. before the study received the same dose of PP3M in the maintenance phase. Early in the study (before the PP3M pre-randomization target was met), participants who received PP1M as 100 or 150 mg eq. before the study or during the transition phase received PP3M as 350 or 525 mg eq. in the maintenance phase. Later in the study (after the PP3M pre-randomization target was met), participants who received PP1M as 100 or 150 mg eq. before the study or during the transition phase again received PP1M as 100 or 150 mg eq. in the maintenance phase.

To appropriately balance the number of PP1M and PP3M participants in the maintenance phase and to complete the study in a timely manner, the pathway to study enrollment that included participants who entered the study with previous PP3M stability was open for only a limited time. The sponsor informed the Interactive Web Response System (IWRS) vendor that the PP3M pre-randomization target was met on 14 September 2018 after which this pathway was closed to enrollment.

For the DB phase, the active control group administered the OL PP1M doses (100 or 150 mg eq.) was converted to DB PP3M doses (350 or 525 mg eq.). The OL PP3M doses (350 or 525 mg eq.) continued at the same DB dose level.

For the investigational drug group, the OL 100 mg eq. PP1M and 350 mg eq. PP3M doses were converted to DB 700 mg eq. PP6M doses. The OL 150 mg eq. PP1M and 525 mg eq. PP3M doses were converted to DB 1000 mg eq. PP6M doses.

### Randomization and blinding

Randomization and blinding were not used for the Transition or Maintenance Phases.

At entry into the DB phase, participants who had received a moderate dose in the maintenance phase were randomly assigned to 1 of 2 treatment groups (active control or investigational drug as moderate doses) and participants who had received a higher dose in the maintenance phase were randomly assigned to 1 of 2 treatment groups (active control or investigational drug as higher doses), based on a computer-generated randomization schedule. The randomization (2:1 ratio, PP6M:PP3M) was balanced by using randomly permuted blocks and was stratified by study site and by moderate or high dose in the maintenance phase. Central randomization was implemented in this study. The IWRS assigned a unique treatment code for treatment assignment and matching study drug kit for the participant. The investigator was not provided with randomization codes. The codes were maintained within the IWRS, which had the functionality to allow the investigator to break the blind for an individual participant for reasons related to the participant’s safety. Data that may potentially unblind the treatment assignment (ie, study drug serum concentrations or prolactin levels) was to be handled with special care to ensure that the integrity of the blind was maintained and to minimize bias. Due to differences in syringe sizes for PP6M versus PP3M, the study drug administrator was unblinded and not allowed to perform any other study-related procedures or communicate participant-related information with study-site personnel to ensure integrity of the blind. Under normal circumstances, the blind was not to be broken until all participants completed the study and the database was finalized. Otherwise, the blind was to be broken only if specific emergency treatment would be dictated by knowing the treatment status of the participant.

### Open-label extension

The primary objective of the subsequent single-arm, 24-month, OL extension (OLE) study (Protocol R092670PSY3016; NCT 04072575) was to assess (in a limited number of countries who participated in the main study) the long-term safety and tolerability of PP6M (700 or 1000 mg eq.) and to provide access to PP6M in participants with schizophrenia who completed the main study without relapse. The initial dose of PP6M received during this OLE (ie, at Visit 1) was determined based on the participant’s dose level (moderate or higher) during the DB phase of the main study. Because treatment assignment (ie, to PP6M vs PP3M) during the DB phase of the main study was blinded when Visit 1 occurred, the dose was fixed for all participants at this visit to ensure that participants treated with PP3M during the DB phase of the main study would initiate treatment with PP6M at an equivalent dose. For participants who did not relapse or withdraw from the study, participation continued for up to a maximum of 2 years. Safety assessment was based on reported AEs mental status examination/clinical assessment, clinical laboratory tests, vital sign measurements, physical examinations, extrapyramidal symptom rating scales, evaluation of the injection site, Columbia Suicide Severity Rating Scale, and ECG findings. Descriptive statistics of the change from baseline (OLE baseline and the baseline of the DB phase of the main study) were also provided.

### Post-hoc data analysis

The intent-to-treat (ITT)-DB analysis set included all randomized participants who received ≥1 dose of treatment (PP3M or PP6M) during the DB phase. The mean change (±standard deviation [SD]) in BW and abnormal weight percent change from baseline were calculated at 6 months, and 12 months by age (18-25 years; 26-50 years; 51-65 years; >65 years), gender, and BMI (normal: <25; overweight: 25 to <30; obese: ≥30 kg/m^2^). The number of participants with abnormal (≥7%) weight percent change from baseline to endpoint was compared between PP3M and PP6M by chi-squared test. Additionally, treatment-emergent shifts for the four key lipid parameters (fasting low density lipoprotein [LDL], fasting triglycerides [TG], fasting total cholesterol [TC], and fasting high density lipoprotein [HDL]) over time were assessed. For participants who enrolled in the OLE, the mean change in BW, the percent change in BW, and the change in BMI from the DB baseline (in the main study) to the end of the OLE study were also calculated.

## Results

Of the 838 participants enrolled and dosed in the OL phases, 702 (83.8%) completed the OL phases and were randomized (PP6M, n = 478; PP3M, n = 224) in the DB phase. A full description of the sample including demographic and baseline characteristics, psychiatric history, and psychopathology measures (PANSS-total score, Personal Social Performance score, and Clinical Global Impression – Severity score) has been described previously.^26^ Additionally, a complete listing of all concomitant medications can be found within the supplementary materials of the primary publication (https://academic.oup.com/ijnp/article/25/3/238/6427395#supplementary-data).

### Abnormal weight (study population) – percent change from DB baseline to end of DB phase

At the start of the DB phase, of the 224 participants treated with PP3M, 33.9% (n = 76) participants had a BMI classified as normal, 36.6% (n = 82) had a BMI classified as overweight, and 29.5% (n = 66) participants had a BMI classified as obese. Of the 478 participants treated with PP6M, 29.1% (n = 139) participants had a BMI classified as normal, 40.2% (n = 192) participants had a BMI classified as overweight, and 30.8% (n = 147) participants had a BMI classified as obese. The number of participants with a significant (defined as a ≥ 7%) increase or decrease in weight change from DB baseline to the end of the 12-month DB phase is shown in Table 1. Of the 224 participants treated with PP3M in the DB phase, 219 (97.8%) reported a change in weight from baseline to end of study. Of these, 15 (6.8%) reported a decrease of ≥7% change from baseline and 29 (13.2%) reported an increase of ≥7%. Across the population, the mean % change from baseline was 2.1% and ranged from a decrease of 21% to an increase of 40.7%. Similarly, of the 478 participants treated with PP6M during the DB phase, 473 (99.0%) reported a change in weight from baseline to the end of the study. Of these, 43 (9.1%) reported a decrease of ≥7% and 50 (10.6%) reported an increase of ≥7% over this period. The proportion of participants reporting an increase of ≥7% change did not significantly differ between participants treated with PP3M and PP6M (*P* = .30), nor did the proportion of participants who reported a decrease of ≥7% (*P* = .31). Across the population, the mean % change from baseline was 1.02% and ranged from a decrease of 29.3% and an increase of 39.9%. For both groups, most of the participants had their weight remain stable throughout the study or fluctuate between ±6.9% from their weight at baseline.

**Table 1:** Number of participants with abnormal weight: percent change from study baseline to double-blind end point.

	**PP3M (N = 224)**	**PP6M (N = 478)**
**Weight percent change**	219	473
**Decrease ≥7%**	15 (6.8%)	43 (9.1%)
**Pearson’s Chi-Square (*P*-value)**		0.31
**Increase ≥7%**	29 (13.2%)	50 (10.6%)
**Pearson’s Chi-Square (*P*-value)**		0.30

Abbreviations: N = number of participants; PP3M = Paliperidone palmitate 3-month formulation; PP6M = Paliperidone palmitate 6-month formulation.

### Body weight by age group – absolute change from DB baseline to end of DB phase

Table 2 shows the mean (±SD) BW of participants at DB baseline and the absolute change in weight from DB baseline to the end of the DB phase, stratified by age group (18-25; 26-50; 51-65; and >65 years of age), for participants treated with PP3M and PP6M. It also shows the number of participants who had a significant (≥7%) increase or decrease in BW over the study period. As shown in Table 2, the greatest weight change from DB baseline occurred in the >65 years age group who had been treated with PP3M. However, this group consisted of only 3 participants, which could lead to potential bias in the results due to the small sample size. Otherwise, the youngest age group (18-25 years of age) who were treated with PP3M (n = 21) showed the greatest increase in mean weight during the DB phase. In this age group the mean (±SD) BW at DB baseline was 78.29 (±15.21) kg and the mean change from baseline to the end of the DB phase was 4.33 (±7.11) kg and 6 (28.6%) participants had an increase of ≥7% in BW from DB baseline to the end of the study. In contrast, participants within this age group who were treated with PP6M (n = 44) showed the greatest decrease in weight from DB baseline to end of the DB phase. A total of seven (15.9%) participants had a decrease of ≥7% in BW from DB baseline to the end of the study. In this age group, the mean (±SD) weight at DB baseline was 77.74 (±15.56) kg and the mean change from DB baseline to the end of the DB phase was –0.65 (±4.96) kg.

**Table 2:** Body weight by age group – change from double-blind baseline to double-blind end point.

	**PP3M (N = 224)**	**PP6M (N = 478)**
**18-25**		
**Weight (kg)**		
**N**	21	44
**Mean baseline (SD)**	78.29 (15.209)	77.74 (15.557)
**Mean change (SD)**	4.33 (7.112)	–0.65 (4.955)
**Decrease ≥7%**	1 (4.8%)	7 (15.9%)
**Increase ≥7%**	6 (28.6%)	5 (11.4%)
		
**26-50**		
**Weight (kg)**		
**N**	160	326
**Mean baseline (SD)**	81.55 (16.893)	83.14 (16.993)
**Mean change (SD)**	0.91 (4.600)	0.29 (4.878)
**Decrease ≥7%**	9 (5.6%)	25 (7.7%)
**Increase ≥7%**	20 (12.5%)	34 (10.4%)
		
**51-65**		
**Weight (kg)**		
**N**	35	95
**Mean baseline (SD)**	80.91 (15.495)	82.91 (16.978)
**Mean change (SD)**	–1.20 (4.763)	–0.31 (5.247)
**Decrease ≥7%**	5 (14.3%)	10 (10.5%)
**Increase ≥7%**	1 (2.9%)	8 (8.4%)
		
**>65**		
**Weight (kg)**		
**N**	3	8
**Mean baseline (SD)**	87.03 (32.362)	73.30 (10.702)
**Mean change (SD)**	5.47 (5.707)	1.76 (4.738)
**Decrease ≥7%**	0	1 (12.5%)
**Increase ≥7%**	2 (66.7%)	3 (37.5%)

Abbreviations: DB = Double-blind; N = number of participants; PP3M = Paliperidone palmitate 3-month formulation; PP6M = Paliperidone palmitate 6-month formulation; SD = Standard deviation.

Results are for participants with both baseline (DB) and end point (DB) data.

### Body weight by BMI classification – absolute change from DB baseline to end of DB phase

As shown in Table 3, for participants with DB baseline BMI in the normal or overweight range, the mean change in BW from DB baseline to the end of the DB phase was smaller for the group of participants treated with PP6M when compared with the group of participants treated with PP3M. It also shows the number of participants who had a significant (≥7% change in BW) increase or decrease over the study period. The largest difference in mean (±SD) change in BW from DB baseline until the end of the DB phase was for the overweight group (1.15 ± 4.81 kg versus –0.53 ± 4.39 kg for participants treated with PP3M and PP6M, respectively) and 4 (5.3%) participants and 21 (11.4%) had an decrease of ≥7% in BW from DB baseline to the end of the study while 7 (9.3%) and 13 (7%) had an increase of ≥7% for PP3M and PP6M, respectively. For participants who were classified as obese at baseline, the mean (±SD) change in BW from baseline was <1 kg regardless of which formulation the participants had been treated with but was smaller for the PP3M group (0.30 ± 5.96 kg versus 0.71 ± 6.45 kg for participants treated with PP3M and PP6M, respectively).

**Table 3:** Body weight by BMI (double-blind baseline) – change from double-blind baseline to double-blind end point.

	**PP3M (N = 224)**	**PP6M (N = 478)**
**NORMAL <25**		
**Weight (kg)**		
**N**	73	133
**Mean baseline (SD)**	65.20 (8.973)	66.14 (8.963)
**Mean change (SD)**	1.42 (4.456)	0.28 (3.404)
**Decrease ≥7%**	3 (4.1%)	7 (5.3%)
**Increase ≥7%**	14 (19.2%)	11 (8.3%)
		
**OVERWEIGHT 25 to <30**		
**Weight (kg)**		
**N**	75	185
**Mean baseline (SD)**	81.93 (10.788)	80.66 (9.342)
**Mean change (SD)**	1.15 (4.814)	-0.53 (4.386)
**Decrease ≥7%**	4 (5.3%)	21 (11.4%)
**Increase ≥7%**	7 (9.3%)	13 (7.0%)
		
**OBESE ≥30**		
**Weight (kg)**		
**N**	71	155
**Mean baseline (SD)**	96.90 (11.898)	98.49 (14.294)
**Mean change (SD)**	0.30 (5.955)	0.71 (6.448)
**Decrease ≥7%**	8 (11.3%)	15 (9.7%)
**Increase ≥7%**	8 (11.3%)	26 (16.8%)

Abbreviations: DB = Double-blind; N = number of participants; PP3M = Paliperidone palmitate 3-month formulation; PP6M = Paliperidone palmitate 6-month formulation; SD = Standard deviation.

Results are for participants with both baseline (DB) and end point (DB) data.

### Body weight by sex – weight percent change from DB baseline to end of DB phase

As shown in Table 4, the percentage of participants with a significant increase or decrease in BW from DB baseline to the end of the DB phase was also analyzed by sex. The number of women with a significant (≥7%) increase or decrease in BW was similar; 10 (14.7%) women treated with PP3M exhibited a ≥ 7% increase from BW compared with 23 (15.2%) of women treated with PP6M. For men, 27 (8.4%) of men treated with PP6M reported a significant (≥7%) increase in BW compared with 19 (12.6%) of men treated with PP3M.

**Table 4:** Body weight by Sex – Weight percent change from double-blind baseline to double-blind end point.

	**PP3M (N = 224)**	**PP6M (N = 478)**
**Women**		
**Weight percent change(kg)**	68	151
**Decrease ≥7%**	9 (13.2%)	11 (7.3%)
**Increase ≥7%**	10 (14.7%)	23 (15.2%)
		
**Men**		
**Weight percent change(kg)**	151	322
**Decrease ≥7%**	6 (4.0%)	32 (9.9%)
**Increase ≥7%**	19 (12.6%)	27 (8.4%)

Abbreviations: DB = Double-blind; N = number of participants; PP3M = Paliperidone palmitate 3-month formulation; PP6M = Paliperidone palmitate 6-month formulation; SD = Standard deviation.

Results are for participants with both baseline (DB) and end point (DB) data.

### Body weight, BMI, waist circumference (study population) – absolute change from DB baseline to end of DB phase

The mean (±SD) change from DB baseline to the end of the DB phase was calculated for BW, weight percent change, BMI, and waist circumference across the entire population treated with PP3M (n = 224) and the entire population treated with PP6M (n = 478) and is summarized in Table 5. For each of the parameters measured, the participants treated with PP6M had a smaller change from DB baseline when compared with the participants treated with PP3M. From DB baseline to the end of the DB phase, in the PP6M and PP3M groups, respectively, the mean (±SD) change in BW was 0.10 (±4.96) kg versus 0.96 (±5.10) kg, the mean percent change in BW was 0.19% (±5.66%) versus 1.33% (±6.20%), the mean (±SD) change in BMI was 0.0 (±1.72) kg/m^2^ versus 0.3 (±1.78) kg/m^2^, and the mean (±SD) change in waist circumference was 0.37 (±5.16) cm versus 0.82 (±5.14) cm.

**Table 5:** Body weight, Body Mass Index, and waist circumference – change from double-blind baseline to double-blind end point.

	**PP3M (N = 224)**	**PP6M (N = 478)**
**Body mass index (kg/m** ^**2**^ **)**		
**N**	219	473
**Mean baseline (SD)**	27.6 (4.94)	28.1 (5.00)
**Mean change (SD)**	0.3 (1.78)	0.0 (1.72)
		
**Waist Circumference (cm)**		
**N**	219	473
**Mean baseline (SD)**	95.71 (13.737)	95.95 (14.972)
**Mean change (SD)**	0.82 (5.137)	0.37 (5.157)
		
**Weight (kg)**		
**N**	219	473
**Mean baseline (SD)**	81.21 (16.667)	82.42 (16.848)
**Mean change (SD)**	0.96 (5.103)	0.10 (4.959)
		
**Weight percent change (%)**		
**N**	219	473
**Mean baseline (SD)**	81.21 (16.667)	82.42 (16.848)
**Mean change (SD)**	1.33 (6.202)	0.19 (5.658)

Abbreviations: DB = Double-blind; N = number of participants; PP3M = Paliperidone palmitate 3-month formulation; PP6M = Paliperidone palmitate 6-month formulation; SD = Standard deviation.

Results are for participants with both baseline (DB) and end point (DB) data.

### Fasting lipids (study population): treatment emergent shifts from DB baseline to end of DB phase

Four lipid parameters during the fasting state (TC, HDL, LDL, and TG) were collected and assessed over time. Treatment-emergent shifts from DB baseline through the end of the DB phase were calculated by treatment group PP3M and PP6M. These results are summarized in Table 6.

**Table 6:** Fasting lipids: treatment-emergent shifts from double-blind baseline during the double-blind phase.

	**PP3M (N = 224)**	**PP6M (N = 478)**
**Fasting Cholesterol (mg/dL)**	194	423
**<200 mg/dL to ≥240 mg/dL**	2 (1.0%)	3 (0.7%)
		
**Fasting HDL Cholesterol (mg/dL)**	194	423
**≥40 mg/dL to <40 mg/dL**	28 (14.4%)	55 (13.0%)
		
**Fasting LDL Cholesterol (mg/dL)**	194	423
**<100 mg/dL to ≥160 mg/dL**	1 (0.5%)	2 (0.5%)
		
**Fasting Triglycerides (mg/dL)**	194	423
**<150 mg/dL to ≥200 mg/dL**	22 (11.3%)	22 (5.2%)

Abbreviations: DB = Double-blind; HDL = High density lipoproteins; LDL = Low density lipoproteins; N = number of participants; PP3M = Paliperidone palmitate 3-month formulation; PP6M = Paliperidone palmitate 6-month formulation; SD = Standard deviation.

For each fasting parameter, participants with both Baseline (DB) record and any post baseline (DB) record during DB Phase are included in the denominator.

For TC, a similar percentage of participants treated with PP3M (1%) and PP6M (0.7%) had shifts from <200 mg/dL (normal range) at DB baseline to ≥240 mg/dL at the end of the DB phase. In addition, the percentage of participants with shifts in LDL cholesterol from <100 mg/dL (normal range) to ≥160 mg/dL was the same in the PP3M and PP6M groups (0.5% in each). However, for TGs, a higher percentage of participants treated with PP3M versus PP6M had a shift from <150 mg/dL (normal range) to ≥200 mg/dL (11.3% vs 5.2%, respectively). Lastly, the percentage of participants with shifts in HDL cholesterol from <40 mg/dL to ≥40 mg/dL was similar in participants treated with PP3M (14.4%) and PP6M (13%).

### Body weight, BMI, waist circumference (study population) – change over time from DB baseline to end of extension study (24 months) by previous treatment group

To determine the long-term effect of PP3M and PP6M on metabolic parameters, BW over time was calculated for participants who had been treated with PP3M in the DB phase and then switched to treatment with PP6M in the OLE (PP3M/PP6M population). Changes in BW were also calculated over time for participants who were treated continuously (up to 36 months) with PP6M. A total of 702 participants were randomized to the DB phase and 178 of these participants completed the DB phase and enrolled in the OLE study. Of the 178 participants who entered the OLE, 35.4% of participants had BMI classified as overweight, and 41.6% had BMI classified as obese. The mean (±SD) weight for the PP3M/PP6M population was 80.28 (±16.27) kg at DB baseline. This increased to 81.70 (±15.27) kg at the end of the OLE. The mean change from baseline at the end of the OLE was 1.61 (±6.18) kg, and the range of change was –15 kg to 20.8 kg. The mean (±SD) percent change in BW (from DB baseline through the end of the OLE) for this population was 2.73% (±8.52%) and ranged from –15.5% to 31.3% change. For participants treated continuously with PP6M, the mean (±SD) weight was 84.42 (±16.38) kg at DB baseline. This increased to 85.25 (±15.87) kg at the end of the 24-month OLE. The mean change from baseline at the end of the OLE was 0.86 (±6.6) kg, and the range of change was –26.6 kg to 21.9 kg. The mean (±SD) percent change in BW for this population was 1.45% (±7.79%) and ranged from –27.7% to 28.3% change.

When BMI was calculated, the mean (±SD) BMI for the PP3M/PP6M population was 27.1 (±4.85) kg/m^2^ at DB baseline. This increased to 27.70 (±4.74) kg/m^2^ at the end of the 24-month OLE. The mean change from DB baseline BMI at the end of the OLE was 0.60 (±2.29) kg/m^2^, and the range of change was –6 kg/m^2^ to 7 kg m^2^. For participants treated continuously with PP6M, the mean (±SD) BMI was 28.4 (±4.91) kg/m^2^ at DB baseline. This increased slightly to 28.6 (±5.00) kg/m^2^ at the end of the 24-month OLE. The mean change from baseline at the end of the OLE was 0.2 (±2.22) kg/m^2^, and the range of change was –8 kg/m^2^ to 7 kg/m^2^.

## Discussion

The current analysis was designed to examine the effect that PP6M had on metabolic parameters including BW, and blood lipid profiles, relative to PP3M, by assessing changes in BW from baseline to the end of study based on age, BMI, and changes in blood lipid profiles during a 12-month clinical study. This analysis also aimed to establish the long-term effects of PP6M on BW and BMI over a 24-month extension study when participants were treated exclusively with PP6M.

Results of this study showed that overall, fewer participants treated with PP6M experienced abnormal weight gain (≥7% from baseline) over the 12-month study period, and more participants (treated with PP6M) experienced a significant decrease in weight during this time. When these data were stratified by age groups, participants between the ages of 18-25 who were treated with PP3M showed the greatest increase in weight over the 12-month period, and those who were treated with PP6M had the greatest reduction in weight during this time. These findings suggest that most participants treated with PP6M, had a more favorable change in weight over the study period (ie, more participants lost weight and fewer participants gained weight), and that PP6M had a better metabolic profile in terms of abnormal weight changes, particularly in adolescent and young adult participants. Results from this study also showed that participants with a BMI that was classified as normal or overweight at baseline exhibited smaller changes in BW over the study period when they were treated with PP6M, relative to those participants with a normal or overweight BMI classification who were treated with PP3M.

It is of interest to note that most of the participants who enrolled in the study were overweight at baseline, with 30.6% of the study population classified as normal weight. However, when the proportion of adults (across the general population) who were classified as normal weight from each of the countries that recruited the largest number of participants was considered, the study’s weight distribution did not differ significantly compared with the general population. For instance, 27.6% of adults are classified as normal weight in the US, 31.6% in Argentina, 37% in Brazil, 37.3% in Poland, 41.2% in Russia, and 43.9% of adults in Ukraine are classified as normal weight compared with 30.6% of adults in this study.^28^ Additionally, prior to study entry, 99.9% of participants enrolled in the OL phase had received ≥1 psychotropic medication. The majority (≥50%) of these psychotropic medications were atypical antipsychotics (62.6% participants in the OL phase were administered atypical antipsychotics prior to enrolment) with oral risperidone being the most common atypical antipsychotic used (30% of participants). Paliperidone was used as a depot antipsychotic in 36.3% of participants prior to the OL phase. Therefore, it is possible that the increase in weight and BMI observed in the study was due to the prior medications administered and not solely due to the administration of the PP3M or PP6M.

Results from this study also showed that overall, the absolute change in BW, BMI, and waist circumference was smaller for participants treated with PP6M, relative to those treated with PP3M. Furthermore, when shifts in fasting lipid parameters were examined, a greater proportion of participants treated with PP3M had shifts in TG levels that were outside the normal range, than those participants who were treated with PP6M. Collectively, these results suggest that the consistent serum concentration of paliperidone palmitate over the course of 6 months allowed for metabolic regulation of BW and fasting lipids, thereby resulting in favorable BMI, waist circumference, and % abnormal weight gain over the course of treatment with PP6M.

Perhaps the most insightful result from this study was the finding that participants who were treated continuously with PP6M for up to 36 months had a lower mean % change in BW over the course of treatment than participants who were initially treated with PP3M (for 12 months) and then switched to PP6M. This lends further support to the hypothesis that consistent exposure to paliperidone palmitate for longer dosing intervals contributes to an overall regulation of metabolic function in these patients.

A notable strength of this post-hoc analysis was that it incorporated data from a 12-month, DB, controlled study that directly compared the safety and efficacy of PP3M and PP6M, and a 24-month OLE study. These controlled data allowed for direct comparisons between the 2 formulations, and established long-term trends in changes in BW, for participants treated exclusively with PP6M for up to 36-months. The results from this post-hoc analysis suggest that PP6M is comparable to PP3M in terms of metabolic parameters and may have a beneficial effect on weight gain, especially in young patients with schizophrenia.

## Data Availability

The data sharing policy of Janssen Pharmaceutical Companies of Johnson & Johnson is available at https://www.janssen.com/clinical-trials/transparency. As noted on this site, requests for access to the study data can be submitted through Yale Open Data Access [YODA] Project site at http://yoda.yale.edu.
